# The Effect of Ge Addition on the Oxidation of Nb-24Ti-18Si Silicide Based Alloys

**DOI:** 10.3390/ma12193120

**Published:** 2019-09-25

**Authors:** Zifu Li, Panos Tsakiropoulos

**Affiliations:** Department of Materials Science and Engineering, The University of Sheffield, Sir Robert Hadfield Building, Mappin Street, Sheffield S1 3JD, UK; li.zi.fu@hotmail.com

**Keywords:** Nb-silicide based alloys, high entropy alloys, complex concentrated alloys, oxidation, intermetallics, silicides

## Abstract

In this paper, we report research about the isothermal oxidation in air at 800 and 1200 °C for 100 h of the as-cast alloys (at.%) Nb-18Si-5Ge (ZF1), Nb-18Si-10Ge (ZF2), Nb-24Ti-18Si-5Ge (ZF3), Nb-24Ti-18Si-5Cr (ZF4), Nb-24Ti-18Si-5Al (ZF5), Nb-24Ti-18Si-5Al-5Cr-5Ge (ZF6), and Nb-24Ti-18Si-5Al-5Cr-5Ge-5Hf (ZF9), the microstructures of which were reported in previous publications. Only the alloys ZF1, ZF2, and ZF3 suffered from pest oxidation at 800 °C. The Ge addition substantially improved the oxidation resistance of the other alloys both at 800 °C and 1200 °C, which followed parabolic kinetics at 800 °C and in the early stages at 1200 °C and linear kinetics at longer times, where there was spallation of the scales. The Nb_2_O_5_ and TiNb_2_O_7_ oxides were formed in the scales of the alloys ZF4, ZF5, ZF6, and ZF9 at 800 °C and 1200 °C, the GeO_2_ was observed in all scales and the SiO_2_ in the scales of the alloys ZF4 and ZF5, the CrNbO_4_ in the scales of the alloys ZF4, ZF6, and ZF9 and the AlNbO_4_ in the scales of the alloys ZF5, ZF6, and ZF9. Diffusion zones were formed below the scale/substrate interface only in the alloys ZF4 and ZF5 where the Nb_ss_ and Nb_5_Si_3_ were contaminated by oxygen. However, these phases were not contaminated by oxygen in the bulk of the alloys ZF4, ZF5, ZF6, and ZF9. The alloys ZF9 and ZF6 exhibited the best oxidation behaviour at 800 °C and 1200 °C, respectively. The alloys were compared with Nb-silicide based alloys of similar compositions without Ge and Hf additions and the alloy ZF9 with refractory metal High Entropy Alloys. Compared with the former alloys, the addition of Ge reduced the vol % of the Nb_ss_. Compared with the latter alloys, the superior oxidation behaviour of the alloy ZF9 was attributed to its higher and lower values respectively of the parameters δ and VEC.

## 1. Introduction

Niobium silicide based alloys are candidate materials to replace Ni-based superalloys in gas turbine engines in the beyond the Ni superalloys era because they have the potential to offer a balance of properties that will make them suitable for structural applications at T > 1200 °C. The basis for the development of these new alloys has been the Nb-Si-Ti-Al-Cr-Hf system. Properties of alloys with additions of other transition, refractory, and simple metals [1,2,3] have moved closer to property targets. The microstructures of the new alloys usually contain a bcc Nb_ss_, and tetragonal and/or hexagonal Nb_5_Si_3_. Tetragonal or metastable Nb_3_Si, C14-NbCr_2_ Laves phase, and other intermetallics also can be present [4,5,6]. The chemistry of the solid solution depends critically on the overall alloy constitution. In most cases the Nb_ss_ is rich in Ti, Al, Cr, and Hf and its Si content is generally low and depends on the synergy of refractory metals with other alloying additions [4,7]. The Nb_ss_ undergoes selective oxidation compared with the Nb_5_Si_3_ silicide and the oxidation resistance of the latter decreases above 1100 °C [8]. Like the Ni-based superalloys, the Nb-silicide based alloys (i) will require coatings to enable them to operate in environments where turbine entry temperatures could approach 1830 °C to meet stringent requirements imposed by operational performance and environmental regulations and (ii) should have some inherent oxidation resistance to survive in case of coating failure. 

Oxide scales will form on any alloy that is exposed to a reactive environment at elevated temperature(s). Stable protective scales are desirable for alloys used in structural applications at high temperatures. A scale can be damaged during service, for example, scale cracking and spallation could occur during thermal cycling owing to differences in the values of coefficient of thermal expansion (CTE) between scale and substrate. Thus, healing of the scale via the reformation and growth of the protective oxides is another important requirement [9]. 

Wagner’s model for the transition from internal to external oxidation of an active element, like for example Al in Nb, gives the critical solute concentration that must be exceeded so that the outward diffusive flux of the active element is rapid compared with the inward diffusive flux of oxygen atoms [10]. Important parameters that determine the critical concentration of the active element are the solubility and diffusivity of oxygen, which must be reduced, and the diffusivity of the active element that must be increased. Chromium is expected to reduce the solubility of oxygen. The diffusivity of oxygen is expected to be reduced by Hf that acts as an oxygen scavenger and by Ti and Cr. For example, the addition of 25 at.% Ti to Nb decreases the diffusivity of oxygen by a factor of 20 [8]. The diffusivity of Al in Nb is increased by alloying with Ti as the latter increases the solid solubility of Al in the Nb_ss_. Formation of a continuous Nb_ss_ in the microstructure can be controlled by the type and concentration of alloying additions. 

The oxidation rate constants give an indication of the growth rate of an oxide. The parabolic rate constant is used to rank oxides in terms of the oxidation protection offered by them. Oxidation protection of alloys at T > 1100 °C has been sought via the formation of either Al_2_O_3_ or SiO_2_ scales. The former is more susceptible to degradation during thermal cycling and the latter is more tolerant to the stresses developed during thermal cycling. A glass of high fluidity at low temperatures that can heal cracks is formed when GeO_2_ solute is added in SiO_2_ [11].

Data for parabolic rate constants of various oxides demonstrates the superiority of Al_2_O_3_ and SiO_2_ scales over other oxides [10]. Al_2_O_3_ and SiO_2_ are highly stable and exhibit low diffusivities for cations and anions. Al_2_O_3_ has small deviations from stoichiometry, low point defect concentrations, and high melting point (2050 °C). SiO_2_ is more permeable to oxygen and nitrogen than alumina at high temperature and has a lower melting temperature [12]. The activation energy for diffusion of oxygen in silica is lower than in alumina. Thus, alumina becomes less effective oxidation barrier than silica as temperature is increased. Nevertheless, Al_2_O_3_ is an excellent barrier to penetration by oxygen and grows at a slower rate than silica at temperatures to about 1300 °C. Alumina and/or SiO_2_ scales would be desirable to form on Nb-silicide based alloys. The activity of Al required for the formation of Al_2_O_3_ cannot be provided by the Al concentration in the Nb-silicide based alloys owing to the effect of the latter on mechanical behaviour at low and high temperatures. Formation of SiO_2_ is more likely but depends on alloy microstructure and the formation of Nb_2_O_5_ on the scale (see below).

The maximum temperature of useful resistance to oxidation with different oxide barriers can be estimated from the parabolic rate constants. A useful limit for materials in thin sections is k_P_ ≈ 10^−10^ g^2^·cm^−4^·s^−1^ (weight gain 6 mg·cm^−2^ in 100 h). At this limit, alloys that form chromia as a scale would be useful to 1100 °C (close to the upper limit for Cr base alloys and (high Cr) Ni or Co base alloys). Alumina and silica formers would be useful respectively to 1425 °C and 1750 °C [13]. The maximum useful temperature for Nb-silicide based alloys, the oxidation resistance of which cannot rely on chromia scales, is determined by the melting point of Nb_2_O_5_ based mixed oxides (see below).

In the case of refractory metal intermetallic based alloys, like Nb or Mo silicide based alloys, another important requirement is that the accelerated catastrophic oxidation, known as pest oxidation, that occurs at low temperatures is eliminated [13]. Suppression of pest oxidation in Mo and Nb silicide based alloys is possible via the synergy of specific alloying additions, which control the vol % of the solid solution and lead to stabilisation of specific phases in the alloy microstructure. Depending on the base refractory metal these alloying additions include Al, B, Cr, Ge, Hf, Sn and Ti [4,14].

Pure Nb oxidises following a parabolic law at a short time and then a linear law at longer times. Niobium exhibits high solubility for oxygen and forms three equilibrium oxides, namely NbO, NbO_2_ and Nb_2_O_5_ [12]. At 1000 °C the scale formed on Nb becomes compact and at 1250 °C a coherent but non-adherent scale is formed [15]. In general, Nb alloys oxidise rapidly in air at T > 650 °C. 

Data about the standard free energy of formation of oxides [10,13] shows (a) that NbO is nearly as stable as SiO_2_, (b) that TiO_2_ is more stable than SiO_2_ but less stable than Al_2_O_3_, (c) that HfO_2_ is more stable than Al_2_O_3_, and (d) that there is small difference in stability between Al_2_O_3_ and the niobate NbAlO_4_ [13]. The Nb_2_O_5_ melts peritectically at 1510 °C [12] and is dominant above 500 °C. Complex oxide scales are formed on multi-component Nb-silicide based alloys [4]. The SiO_2_ and Nb_2_O_5_ have no solid solubility. Nb_2_O_5_ in the presence of Al_2_O_3_, SiO_2_ and TiO_2_ forms eutectics with lower than 1510 °C eutectic temperatures (T_m_^Nb2O5 – Al2O3^ = 1400 °C, T_m_^Nb2O5 – SiO2^ = 1448 °C, T_m_^Nb2O5 – TiO2^ = 1467 °C [13]). Nb_2_O_5_ and GeO_2_ form a peritectic at 25 mol % GeO_2_ and 1420 °C, and a eutectic at about 97 mol % GeO_2_ and 1090 °C [16]. GeO_2_ solute in SiO_2_ results in a glass with high fluidity at low temperatures to enable the healing of cracks. The CTE of GeO_2_-SiO_2_ is significantly higher than that of pure SiO_2_ [17]. Formation of metastable solid solution and GeSiO_4_ has been reported by shock wave synthesis, with the latter compound stabilised by excess SiO_2_. Both metastable phases decomposed above 400 °C [18].

In non-Si containing Nb alloys slowest growing oxides are the niobates NbAlO_4_ or NbCrO_4_ (rutile (tetragonal) oxide structures [4,19]). Parabolic oxidation rate constants in the range 7.5 × 10^−10^ to 3 × 10^−9^ g^2^·cm^−4^·s^−1^ have been measured for Nb-Cr-Al alloys, and the intermetallics NbAl_3_ and NbCr_2_ at 1200 °C [19]. The above niobates formed in the scales of the alloys Nb-24Ti-18Si-5Al and Nb-24Ti-18Si-5Cr and other Nb-silicide based alloys [4]. 

The dissolution of oxygen and nitrogen increases the DBTT of the Nb_ss_ which is also hardened and embrittled [20]. These interstitial elements have high permeability in Nb and its alloys and high diffusivities through the base metal oxides [21]. Our research group has shown that contamination by interstitials of Nb-silicide based alloys (a) can lead to the formation of oxides and/or nitrides in their bulk microstructures [22,23] and (b) can be controlled via the synergy of specific alloying additions [24]. In oxidised Nb-silicide based alloys, a so-called diffusion zone develops between the scale and the bulk of the alloy [25]. The thickness of this zone can be controlled by the synergy of alloying additions. The composition of the Nb_ss_ and intermetallics in the diffusion zone is modified compared with the bulk owing to the consumption of specific elements to form the scale [26]. 

The authors have discussed how Ge in synergy with Si and other transition and simple metals affects phase selection and stability as well as the morphology, scale and volume fractions of phases in Nb-silicide based alloys [27,28,29,30,31]. To our knowledge, these studies of Ge containing Nb-silicide based alloys were the first ones to have clarified the role of Ge in the microstructure of Nb-silicide based alloys with the key alloying additions Al, Cr, Hf, and Ti. Ge was also reported to delay breakaway oxidation of Nb- silicide based alloys by at least an order of magnitude in time [32]. Under cyclic and isothermal oxidation conditions, a Ge doped silica film improved the oxidation resistance of Cr-NbCr_2_ based alloys coated with a Ge doped silicide based coating [33]. Germanium doped (Mo,W)(Si,Ge)_2_ coating on a Nb based alloy improved significantly the oxidation of the latter. No pest oxidation was exhibited by the coated alloy or the silicide coating [34], in contrast with the oxidation behaviour of (Mo,W)Si_2_ [35].

Ge doped silicide coating has been applied on Nb-(40,25 and 10)Ti-15Al alloys. The coatings protected the substrate from interstitial contamination. On the Nb-40Ti-15Al alloy a multilayer coating formed with (Nb,Ti)(Si,Ge)2 qnd (Ti,Nb)(Si,Ge)2 outer layer and after cyclic oxidation at 1200 °C, some spallation of the coating was observed [36]. 

The addition of Ge and B improved the protective SiO_2_ phase grown during high-temperature oxidation and resulted in the formation of a continuous and dense oxide scale [37]. The addition of 6 at.% Ge in Nb-22Ti-12Si destabilised the Nb_3_Si, promoted the formation of Nb_5_Si_3_, significantly refined the microstructure and improved the high-temperature strength and room temperature fracture toughness respectively by about 88 and 19%, compared with the alloy without Ge [38]. The fracture toughness (K_C_, 3–point bend) of as-cast and heat-treated Nb-Ti-Si-Cr-Hf-Ge solid solution and silicide based alloys and the indentation toughness of the solid solution, Laves, and silicide phases have been reported [39]. The fracture toughness was not significantly changed by heat treatment and was about 10 MPa·m^1/2^ for alloys with more than 50 vol % silicide and Laves phases while the toughness of the solid solution ranged from 22 to 32 MPa·m^1/2^.

The creep rate of Mo-9Si-8B-*x*Al-*y*Ge (*x*,*y* = 0 or 2) alloys (at.%) at 1200 °C increased when both Al and Ge were present [40]. Soleimani-Dorcheh et. al. [41] studied the oxidation at 1350 °C of Cr–Cr_3_Si alloys with composition Cr_85_Ge*_x_*Si_1−*x*_ (0  <  *x*  <  15). They reported improved oxidation kinetics for up to 2 at % Ge addition, which altered the morphology of locally formed silica and subsequently affected the spallation of chromia scale, and significant weight loss at higher Ge concentrations. The cyclic oxidation of Ge doped Ni-Al-Ti alloys at 1200 °C improved as the Ge concentration increased and GeO_2_ formed in the scales together with NiO, NiTiO_3_, and TiO_2_ [42]. The addition of Ge in Ni-21Cr-2.7Ti-0.9Al-0.05C only significantly improved the spallation of the scale for concentrations less than 2% and the contribution of Ge in the reduction of oxidation rate was significant only for the addition of less than 1%. Weak evidence for GeO_2_ in the scale was reported after 2 and 48 h isothermal oxidation at 1100 °C for the Ni-21Cr-2.7Ti-0.9Al-0.05C-2Ge alloy [43]. 

In this paper, we present the results of our preliminary study of the oxidation of as-cast Ge containing Nb-silicide based alloys the microstructures of which were discussed in [27,28,29,30,31]. Owing to subtle differences between the alloys, the results for each alloy are presented separately. The beneficial effect of Ge in the suppression of pest oxidation at 800 °C will be discussed and it will be shown that Ge in synergy with Al, Cr, and Hf (each at 5 at.% addition) cannot eliminate scale spallation at 1200 °C. The oxidation of Ge containing alloys will be compared with refractory metal (RM) High Entropy Alloys (HEAs) or Complex Concentrated Alloys (CCAs). 

## 2. Experimental

The nominal compositions of the alloys (in this work all compositions are given in at.%) were Nb-18Si-5Ge (ZF1), Nb-18Si-10Ge (ZF2), Nb-24Ti-18Si-5Ge (ZF3), Nb-24Ti-18Si-5Cr-5Ge (ZF4), Nb-24Ti-18Si-5Al-5Ge (ZF5), Nb-24Ti-18Si-5Al-5Cr-5Ge (ZF6), and Nb-24Ti-18Si-5Al-5Cr-5Ge-5Hf (ZF9). The alloys were prepared by arc melting high purity elements (Nb 99.99 wt.%, Ti 99.95 wt.%, Si 99.999 wt.%, Ge 99.999 wt.%, Cr 99.5 wt.%, Hf 99.7 wt.%, and Al 99.999% wt.%) in a copper water-cooled crucible under high purity argon atmosphere with a tungsten electrode. Each alloy was turned over five times to homogenize its composition as much as possible. The microstructures of the alloys were described in [27,28,29,30,31]. Table 1 shows the phases that were observed in the as-cast alloys.

The oxidation of the as-cast alloys was studied using thermo-gravimetric (TG) analysis. The oxidation experiments were done in air at 800 °C and 1200 °C for 100 h using a Pyris 1 TG instrument (PerkinElmer, Shelton, CT, USA) cubic samples of 3 × 3 × 3 mm^3^ volume were cut from the bulk of the cast buttons, and their surfaces were ground to 1200 grit. The dimensions of each sample were measured using a micrometre. Each sample was placed in a small alumina crucible, which was positioned on the TG instrument balance, and then the balance was calibrated to the initial total weight. The sample was heated to the designated temperature and was held at that temperature for 100 h, and then cooled in the furnace. The heating and cooling rates used in the experiments were 3 degrees per min. In each experiment, the mass change versus time was recorded. The mass change was normalised against the initial surface area of the sample. The weight of the sample was measured before and after each TG experiment in order to verify mass change results. 

The scales that formed on the samples used for the TG experiment were studied by glancing angle X-ray diffraction (GXRD, Siemens D5000, Hiltonbrooks Ltd, Crew, UK). The cross-sections of the alloys oxidised at 800 °C were slowly sectioned using a spark erosion cutting machine. Then, the specimens were mounted and polished for imaging and EPMA studies. 

The microstructures of the oxidised alloys were studied using CAMECA SX-50 and SX-51 Electron Microprobe Analysis (EPMA) instruments (CAMECA SAS, Gennevilliers Cedex, France). Samples of high purity Nb, Ti, Si, Ge, Cr, Hf, and Al elements and TiO_2_, Cr_2_O_3_, Al_2_O_3,_ and CaSiO_3_ compounds were ground and polished to 1 μm finish for standardization purpose. A minimum of 10 analyses were performed on phases with size ≥ 5 μm. An accelerating voltage of 20 kV and beam current of 20 nA were used for the analyses. The Phi-Rho-Z correction was used.

A Siemens D5000 X-ray diffractometer with CuKα (λ = 1.540562 Å) radiation was used for GXRD. In this technique, a parallel monochromatic X-ray beam falls on a sample surface at a fixed incidence angle (γ) and the diffraction profile is recorded by a detector. The penetration depth of X–rays in the material depends on the incidence angle (glancing angle), so that when the incidence angle decreases, the diffracted and scattered signals at the angle 2θ arise mainly from a limited depth from the surface, which makes a detection of the phases present in a thin oxide layer possible. In other words, by increasing the glancing angles in an appropriate range, the phases present in various depths in the oxide scales can be detected. 

In order to avoid signal interference from the base alloy (the substrate) the selection of glancing angles was done using the AbsorbDX V1.1.2 software. This software is based on attenuation of X–rays in materials and allows calculation of the depth for most contribution to the diffracted beam. The depth should be smaller than the scale thickness observed in backscatter electron images of the microstructure in a cross-section of the oxidised alloy in order to avoid signal interference from the substrate.

X-ray diffraction of spalled scales was done using powder samples in a Siemens D500 X-ray diffractometer (Hiltonbrooks Ltd, Crew, UK) with CuK_α_ radiation (λ = 1.540562 Å), voltage of 40 kV, current of 30 mA, and a step of 0.02 degrees per second. Identification of the oxides was done using ICDD (International Centre for Diffraction Data) database.

## 3. Results

### 3.1. Oxidation at 800 °C

**Figure 1:**
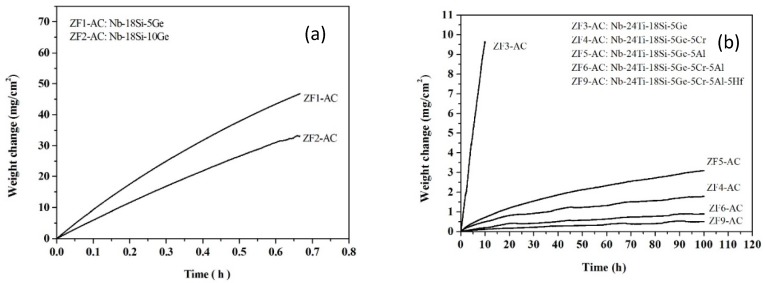
TG curves at 800 °C of the alloys (**a**) ZF1 and ZF2 (effect of Ge addition), (**b**) ZF3 (effect of synergy of Ti and Ge), ZF4 (effect of synergy of Ti, Cr, and Ge), ZF5 (effect of synergy of Ti, Al, and Ge), ZF6 (effect of synergy of Ti, Cr, Al, and Ge), and ZF9 (effect of synergy of Ti, Cr, Al, Ge, and Hf). Notice the different axes used in (**a**) and (**b**).

The TG results are shown in Figure 1, where the mass change per unit area is plotted as a function of time. The total mass change of each alloy is summarised in Table 2 together with the linear oxidation rate constants (k_l_) of the alloys ZF1, ZF2 and ZF3 and the parabolic oxidation rate constants (k_p_) of the alloys ZF4, ZF5, ZF6, and ZF9. The rates were calculated using Equations 1 and 2, respectively [10]

(1) $$\frac{\Delta W}{A}=k_{l}t$$

(2) $${\left(\frac{\Delta W}{A}\right)}^{2}=k_{p}t$$

where ΔW is the mass change of the specimen, A is its surface area prior to oxidation, and t is the exposure time. Compared with the alloy ZF3, the oxidation rates of the alloys ZF4 and ZF6 were five orders of magnitude lower, that of the alloy ZF5 was four orders of magnitude lower, and that of the alloy ZF9 was six orders of magnitude lower.

**Figure 2:**
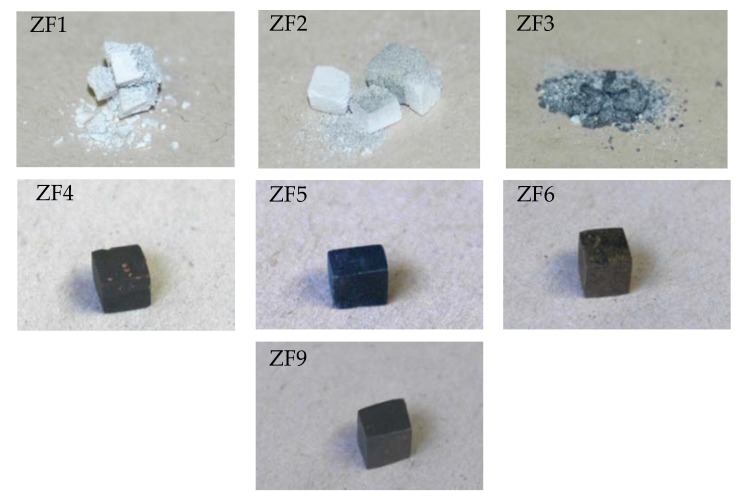
TG samples after isothermal oxidation at 800 °C for 100 h showing that the alloys ZF1, ZF2, and ZF3 experienced pest oxidation, and that formation of thick scales or spallation of the scales was not observed in the alloys ZF4, ZF5, ZF6, and ZF9.

The TG samples after isothermal oxidation are shown in Figure 2. The alloys ZF1, ZF2, and ZF3 disintegrated into powder, i.e., they suffered catastrophic pest oxidation. In contrast, the alloys ZF4, ZF5, ZF6, and ZF9 did not exhibit pest oxidation, and formation of thick scale or spallation of the scale was not observed. 

#### 3.1.1. Structure of Scales, Diffusion Zones and Contamination of Phases by Oxygen

##### Nb-24Ti-18Si-5Ge-5Cr (alloy ZF4)

**Figure 3:**
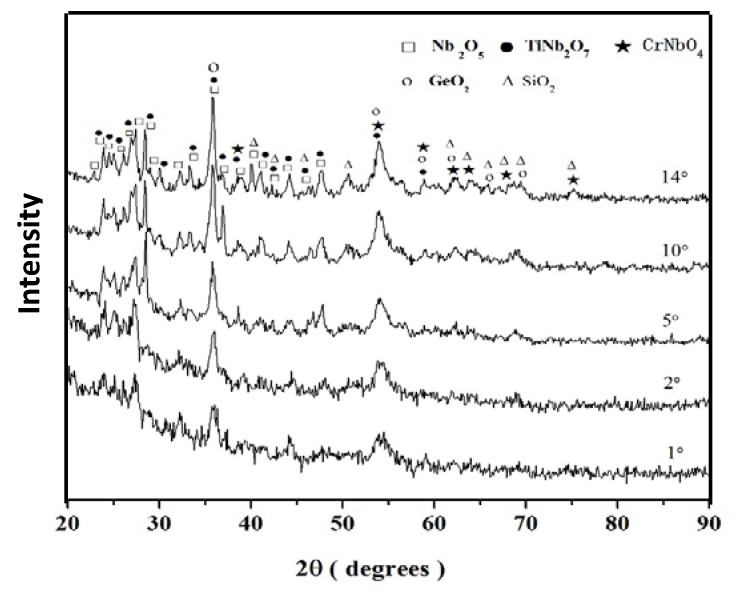
GXRD data (γ = 1°, 2°, 5°, 10°, and 14°) for the scale formed on the alloy ZF4 at 800 °C in air.

The GXRD data (γ = 1°, 2°, 5°, 10°, and 14°) of the scale formed on the alloy ZF4 is shown in Figure 3. The diffraction peaks of the Nb_2_O_5_ and TiNb_2_O_7_ oxides were present in the diffractograms taken at all glancing angles. Most diffraction peaks of the oxides SiO_2_, GeO_2_, and CrNbO_4_ were observed from γ ≥ 5°. At γ = 14° most of the peaks corresponded to the oxides Nb_2_O_5_ and TiNb_2_O_7_, there was only one independent peak for SiO_2_ (2θ = 50.6°), and no independent peak corresponding to the GeO_2_ and CrNbO_4_ oxides.

**Figure 4:**
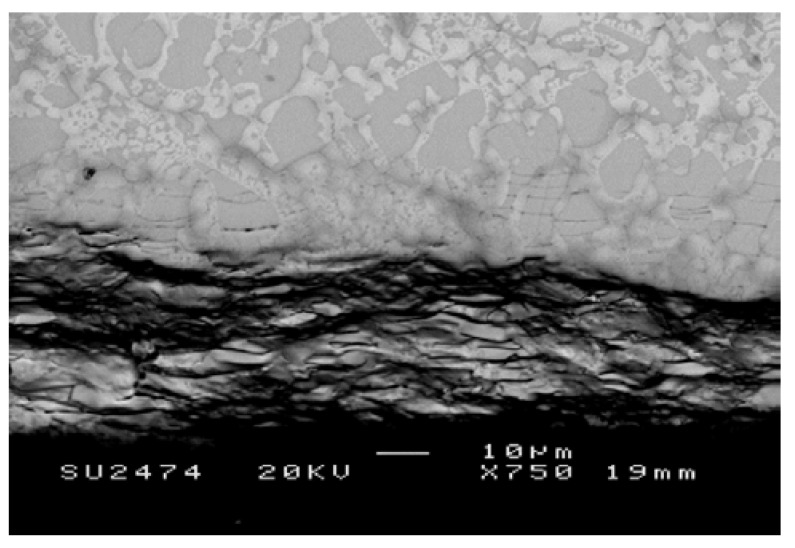
BSE image showing the typical microstructures of a cross-section of the alloy ZF4 after oxidation in air at 800 °C for 100 h.

A cross-section of the alloy ZF4 is shown in Figure 4. Three regions, namely oxide scale, diffusion zone, and bulk alloy could be differentiated by the contrast of the phases. The scale (about 50 μm thick) was severely cracked and porous. In the diffusion zone, which was about 20 μm thick, the Nb_5_Si_3_ exhibited cracks parallel to the surface of the specimen.

Table 3 shows the chemical analysis data of the Nb_ss_ and Nb_5_Si_3_ in the diffusion zone and bulk of the alloy. Oxygen was dissolved in both the Nb_ss_ and Nb_5_Si_3_ (about 21.6 at.% and 2.4 at.%, respectively) in the diffusion zone_,_ but these phases were oxygen-free in the bulk. The oxidised Nb_ss_ exhibited darker contrast under backscatter electron (BSE) imaging conditions compared with that in the bulk. In the Nb_ss_ in the diffusion zone, the concentrations of Nb, Ti, and Ge respectively were lower by 17.7 at.%, 3.4 at.%, and 0.4 at.% than in the bulk. The compositions of the Nb_5_Si_3_ in the two regions were very close. The microstructure in the bulk of the alloy was similar to that in the as-cast alloy but without the C14-Cr_2_Nb Laves phase [29]. 

##### Nb-24Ti-18Si-5Ge-5Al (alloy ZF5) 

**Figure 5:**
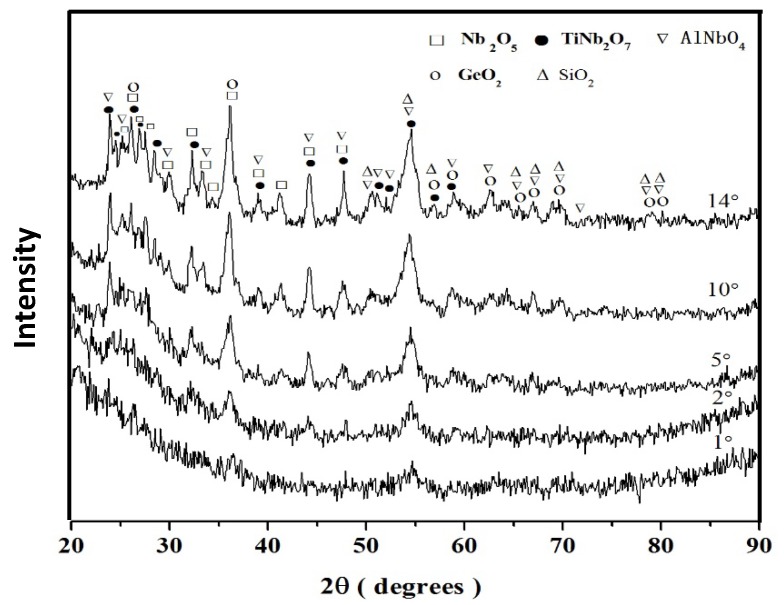
GXRD data (γ = 1°, 2°, 5°, 10°, and 14°) for the scale formed on the alloy ZF5 after oxidation in air at 800 °C.

The GXRD (γ = 1°, 2°, 5°, 10°, and 14°) showed that most diffraction peaks could not be observed at low γ (1° and 2°), but were present with increasing γ (Figure 5). At γ = 14°, the Nb_2_O_5_, TiNb_2_O_7_, AlNbO_4_, SiO_2_ and GeO_2_ oxides were observed. There were characteristic peaks for the former three oxides, and the peaks of SiO_2_ and GeO_2_ overlapped with those of others. Most peaks of the Nb_2_O_5_ and TiNb_2_O_7_ oxides were present at 2θ < 60°, and they were observed from γ ≥ 2°. The peaks for the AlNbO_4_ oxide were found throughout the 2θ angle range, and those of SiO_2_ and GeO_2_ were present at 2θ > 55°. The peaks of the latter two oxides were present at γ values of 5°, 10°, and 14°. 

**Figure 6:**
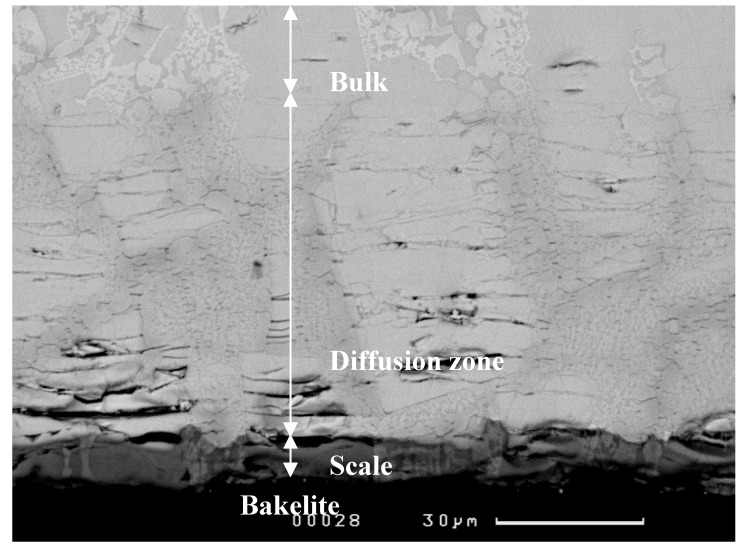
BSE image showing the typical microstructures of a cross-section of the alloy ZF5 after oxidation in air at 800 °C for 100 h.

Figure 6 shows a cross-section of the alloy. Three different regions were observed. The scale and diffusion zone in ZF5 were respectively thinner by about 37 μm (actual average thickness 13 μm) and thicker by about 53 μm (actual average thickness 73 μm) than those in ZF4. The scale of the alloy ZF5 was less cracked and porous compared with the alloy ZF4. In the diffusion zone, the Nb_5_Si_3_ exhibited cracks parallel to the surface of the specimen. 

The oxygen content in the Nb_ss_ and Nb_5_Si_3_ in the diffusion zone was about 32 at.% and 2.6 at.%, respectively (Table 4). These phases were free from oxygen in the bulk. Comparison of the composition of the Nb_ss_ in the diffusion zone with that in the bulk showed that the concentrations of Nb, Ti, Si, Ge, and Al in the former respectively were 17.6 at.%, 11.6 at.% 0.4 at.%, 0.7 at.%, and 1.7 at.% lower than those in the latter. 

##### Nb-24Ti-18Si-5Ge-5Cr-5Al (alloy ZF6)

**Figure 7:**
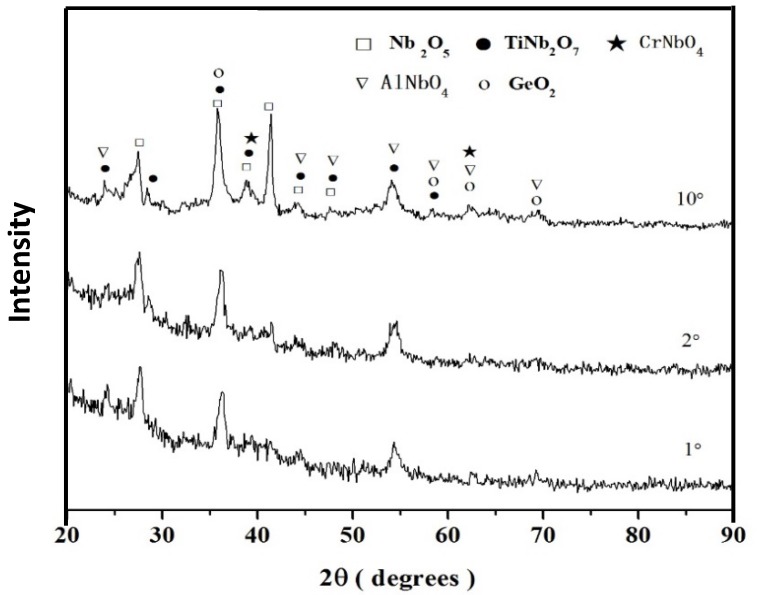
GXRD data (γ = 1°, 2°, and 10°) for the scale formed on the alloy ZF6 at 800 °C in air.

The GXRD (γ = 1°, 2° and 10°) of the scale (Figure 7) showed that the Nb_2_O_5_, TiNb_2_O_7_, AlNbO_4_, CrNbO_4,_ and GeO_2_ oxides were present and that the same diffraction peaks were observed for all angles. There were independent peaks corresponding to the former two oxides, and not for the latter three oxides. Peaks corresponding to SiO_2_ were not observed. 

**Figure 8:**
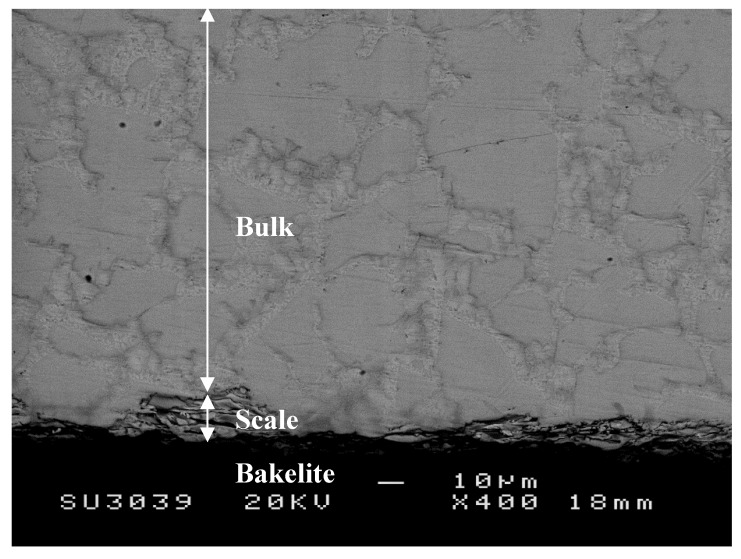
BSE image showing typical microstructures of a cross-section of the alloy ZF6 after oxidation in air at 800 °C for 100 h.

As was the case in the alloy ZF4, the scale was porous and cracked (Figure 8). Its thickness was about 8 μm, thinner than the scale formed on the alloys ZF4 and ZF5. Unlike the latter two alloys, the Nb_ss_ and Nb_5_Si_3_ below the scale were free from oxygen (Table 5), in other words, no diffusion zone was formed. The compositions of these phases were within the range of those observed in the as-cast alloy prior to oxidation [30]. The C14-Cr_2_Nb Laves phase was not observed. 

##### Nb-24Ti-18Si-5Ge-5Cr-5Al-5Hf (alloy ZF9)

**Figure 9:**
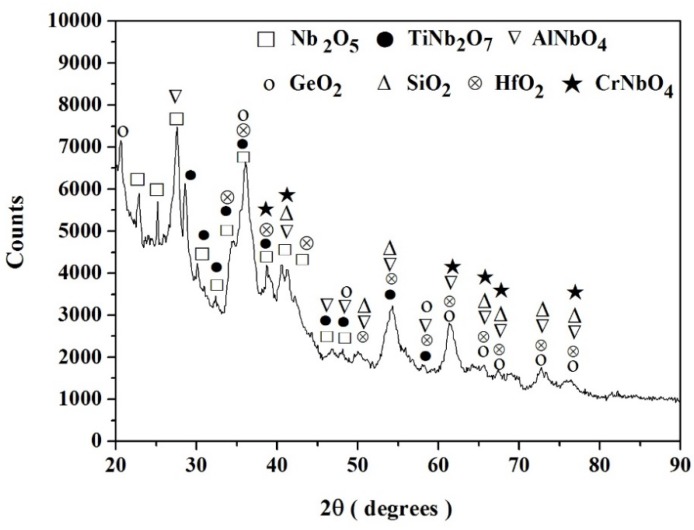
GXRD data (γ = 1°) for the scale formed on the alloy ZF9 after oxidation in air at 800 °C for 100 h.

The GXRD data (γ = 1°) showed that the Nb_2_O_5_, TiNb_2_O_7_, AlNbO_4_, CrNbO_4,_ SiO_2_, GeO_2,_ and HfO_2_ oxides were present (Figure 9). There were peaks corresponding only to the oxides Nb_2_O_5_ and TiNb_2_O_7_. There was also an independent peak corresponding to GeO_2_ (2θ = 20.6) that was not observed in the scales of the alloys ZF4, ZF5, and ZF6. There were no independent peaks for the oxides AlNbO_4_, CrNbO_4_, SiO_2,_ and HfO_2_. 

The thickness of the scale was about 1 μm, which was about 1/50, 1/13 and 1/8 of the thickness of the scales formed on the alloys ZF4, ZF5, and ZF6, respectively. The phases in the microstructure below the scale were oxygen-free (Table 6). 

### 3.2. Oxidation at 1200 °C

**Figure 10:**
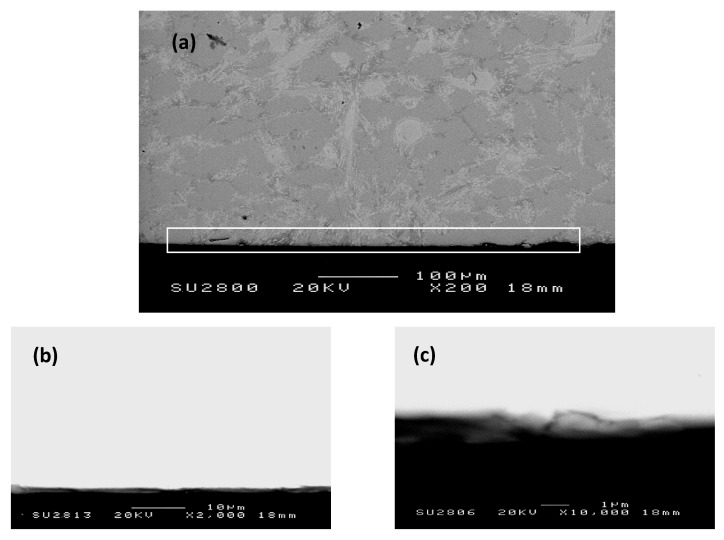
BSE images showing the typical microstructure of a cross-section of the alloy ZF9 after oxidation in air at 800 °C for 100 h: (**a**) ×200, (**b**) ×2000, and (**c**) ×10000.

Owing to the poor oxidation of the alloys ZF1, ZF2, and ZF3 at 800 °C, only the oxidation of the alloys ZF4, ZF5, ZF6, and ZF9 were studied at 1200 °C. The TG data for the isothermal oxidation at 1200 °C of these alloys is shown in Figure 10. All alloys exhibited two-stage oxidation kinetics with the first stage corresponding to parabolic oxidation followed by linear oxidation in the second stage. The oxidation rate constants of each of the aforementioned alloys are shown in Table 7. 

The oxidation of the alloys ZF4 and ZF5 were similar. Parabolic behaviour was observed before 35 h with mass change 29.04 mg·cm^−2^ for the former and 28.42 mg·cm^−2^ for the latter alloy. After 35 h, the oxidation was controlled by linear kinetics in both alloys, and the total mass changes were 59 mg·cm^−2^ and 55 mg·cm^−2^, respectively. In the first oxidation stage, the mass change of the alloy ZF4 was lower than that of the alloy ZF5 before 20 h, then both alloys exhibited the same oxidation behaviour between 20 h and 35 h, and after 35 h the oxidation rate of ZF4 was higher than that of ZF5.

**Figure 11:**
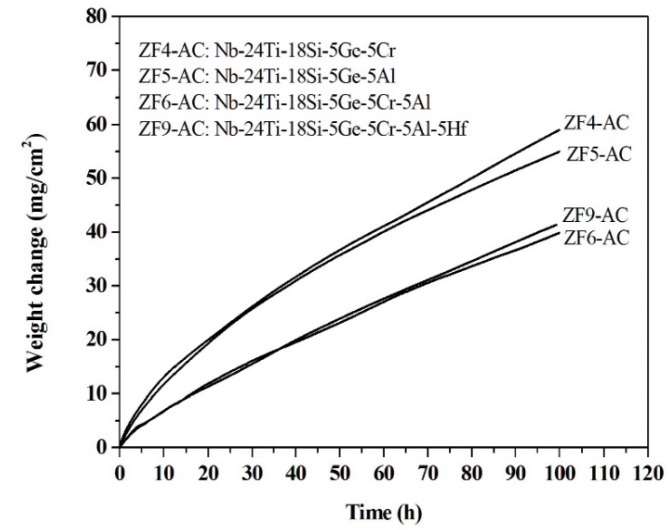
The TG data after isothermal oxidation at 1200 °C for 100 h.

Compared with the alloys ZF4 and ZF5, the oxidation of the alloys ZF6 and ZF9 were better. The oxidation of the latter two alloys was essentially the same (Figure 11), parabolic oxidation was observed before 20 h, and this was followed by linear oxidation. The total mass changes of the alloys ZF6 and ZF9 after 100 h were 39.9 mg·cm^−2^ and 41.4 mg·cm^−2^, respectively. 

**Figure 12:**
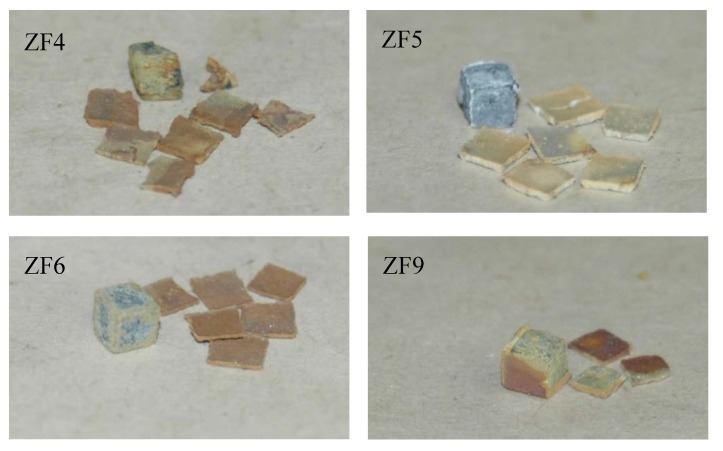
TG samples after isothermal oxidation at 1200 °C for 100 h showing the spallation of the scales.

The TG samples after oxidation are shown in Figure 12. Spallation of the scales was observed for all alloys. in the case of the alloy ZF9, the oxide scale did not separate from three sides of the specimen. The thickness of the spalled scales (Table 8) was significantly larger than the scale that formed at 800 °C (Table 2). The scale formed on ZF6 was the thinnest and was about 42.1%, 42.4%, and 15.1% thinner than that formed on the alloys ZF4, ZF5, and ZF9, respectively and about 49 times thicker than that formed after 100 h at 800 °C. 

**Figure 13:**
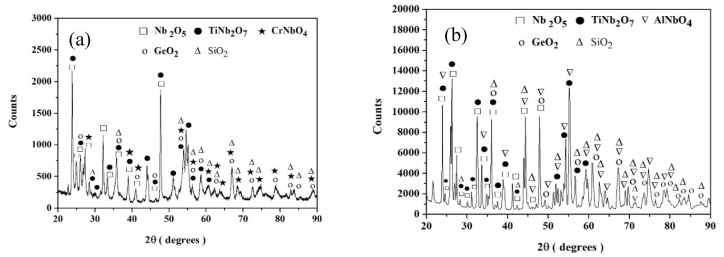

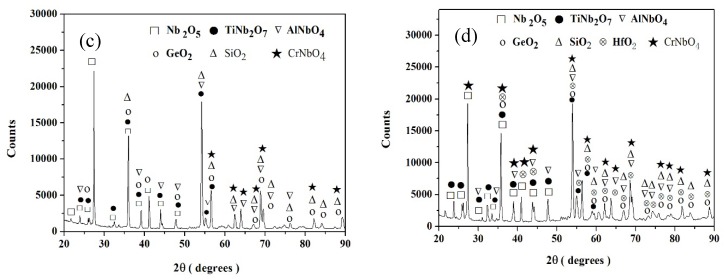
X-ray diffraction data of the spalled scales formed on the alloys (**a**) ZF4, (**b**) ZF5, (**c**) ZF6, and (**d**) ZF9 after 100 h at 1200 °C.

The XRD data for the spalled scales is shown in Figure 13a–d. The oxides in the scales were similar to those formed at 800 °C after 100 h. The Nb_2_O_5_ and TiNb_2_O_7_ were the dominant oxides, and the Cr and Al additions promoted the formation of CrNbO_4_ and AlNbO_4_, respectively. The XRD data indicated the possible presence of the SiO_2_ and GeO_2_ oxides.

## 4. Discussion

### 4.1. Oxidation at 800 °C

#### 4.1.1. Pest Oxidation

Pest oxidation leads to the disintegration of a susceptible compound or alloy into small particles or powder [44]. The alloys ZF1, ZF2, and ZF3 suffered from pest oxidation after exposure at 800 °C from their as-cast condition (Figure 2). The pest oxidation occurred in the alloys ZF1 and ZF2 after 1 h, and in the alloy ZF3 after 10 h. The Ti addition in ZF3 delayed the onset of pest oxidation, as expected because of the beneficial effect of Ti on the oxidation of Nb alloys [25,45] but did not suppress pesting. According to Westbrook and Wood [46], at temperatures in the pest regime oxygen diffuses rapidly through the sample along grain boundaries. In the initial stage, oxidation is confined at the grain boundaries and causes embrittlement. As oxide(s) form(s) internal stresses are generated that cause the disintegration of the specimen along the grain boundaries. At higher temperatures the local hardening and embrittlement are relieved and pest oxidation does not occur. They suggested that the lower temperature limit of pest oxidation is the temperature at which appreciable inter-granular diffusion occurs, while the upper-temperature limit is the temperature at which grain boundary hardening decreases abruptly.

The alloy Nb-24Ti-18Si followed linear oxidation kinetics with k_l_ about 5.6 × 10^−6^ g·cm^−2^·s^−1^ and suffered catastrophic pest oxidation [47]. Comparison of the oxidation of the alloy ZF3 with the latter alloy shows that the 5 at.% Ge addition in Nb-24Ti-18Si-5Ge lowered the oxidation rate constant to 2.7 × 10^−7^ g·cm^−2^·s^−1^, but did not change the oxidation kinetics and that the catastrophic pest oxidation was not eliminated by the synergy of 24 at.% Ti with 5 at.% Ge. Zelenitsas and Tsakiropoulos [47] attributed the pest oxidation of the alloy Nb-24Ti-18Si to the large vol % of the (Nb,Ti)_3_Si silicide (about 64%), the formation of voluminous oxides at the grain boundaries and the generation of inter-granular cracks that increased the oxygen intake and led to accelerated oxidation. The alloy ZF3 contained a large volume fraction of (Nb,Ti)_3_(Si,Ge) (about 79.4% [28]). It is suggested that the mechanism for pest oxidation in ZF3 was essentially the same as for the alloy Nb-24Ti-18Si. 

The alloys ZF4, ZF5, ZF6, and ZF9 did not pest (Figure 2). This would suggest that in the presence of Ti and Ge the additions of Cr and Al either individually or simultaneously suppressed pest oxidation. 

#### 4.1.2. Oxidation Rates

The alloys ZF1 (Nb-18Si-5Ge) and ZF2 (Nb-18Si-10Ge) followed linear oxidation kinetics and exhibited the worst oxidation behaviour with total weight gains of 47.0 mg·cm^−2^ for the former and 33.2 mg·cm^−2^ for the latter after exposure of 0.67 h at 800 °C. The alloy ZF3 (Nb-24Ti-18Si-5Ge) also followed linear oxidation kinetics, but its total weight gain was 9.6 mg·cm^−2^ after exposure of 10 h.

Comparison of the data for the alloys ZF3, ZF4, ZF5, and ZF6 in Table 3 shows that the additions of Cr and Al individually or simultaneously changed the oxidation kinetics from linear to parabolic, which would suggest that in the later three alloys the oxidation process was diffusion controlled. Comparison of the total mass changes after 100 h of ZF4 (1.8 mg·cm^−2^) with Nb-24Ti-18Si-5Cr (95.0 mg·cm^−2^ [47]), ZF5 (3.1 mg·cm^−2^) with Nb-24Ti-18Si-5Al (22.2 mg·cm^−2^ [47]), ZF6 (0.9 mg·cm^−2^) with Nb-24Ti-18Si-5Al-5Cr (34.4 mg·cm^−2^ [47]), Nb-24Ti-18Si-5Al-5Cr-5Mo (20 mg/cm^2^ [25]), and Nb-24Ti-18Si-5Al-5Cr-2Mo (4.0 mg·cm^−2^ [16]), and ZF9 (0.6 mg·cm^−2^) with Nb-24Ti-18Si-5Al-5Cr-5Hf-2Mo (4.7 mg·cm^−2^ [25]) and Nb-24Ti-18Si-5Al-5Cr-5Hf-5Sn-2Mo (1.9 mg·cm^−2^ [48]) shows that Ge substantially improved the oxidation resistance of Nb-silicide based alloys. The mass change of the Hf-containing alloy (ZF9) at 800 °C was the lowest among all the ZF series alloys. 

The mass change of the alloy ZF9 at 800 °C was also lower than that of Nb-26Ti-12.6Si-6.7Cr-5Ge-1.9Al-1.9Hf-0.5Sn-(B,Ce,Fe) (alloy 2 in [32]), which would suggest that the synergy of Ti, Cr, Al, Ge and Hf in ZF9 was more effective than that of these elements with B, Ce, Fe, and Sn in the alloy 2. Furthermore, the oxidation data for the alloys ZF4, ZF5, Nb-24Ti-18Si-5Cr and Nb-24Ti-18Si-5Al [47] would suggest that the synergy of Al with Ge was less effective than that of Cr and Ge in controlling oxidation behaviour at 800 °C.

The parabolic oxidation rate constant of the alloy ZF6 was lower than those of the alloys ZF4 and ZF5. In the alloy Nb-24Ti-18Si-5Cr-5Al, the addition of Al almost doubled the thickness of the diffusion zone and reduced the porosity and cracks in the scale compared with the alloy Nb-24Ti-18Si-5Cr [47] and the scale was thinner compared with that formed on the alloys Nb-24Ti-18Si-5Cr and Nb-24Ti-18Si-5Al [47]. The data for the alloys ZF4, ZF5, and ZF6 would suggest that Al and Cr individually or simultaneously in synergy with Ge reduced further the thickness of the scale and diffusion zone but not the porosity and cracks in the scale. The data for the alloys Nb-24Ti-18Si-5Cr, Nb-24Ti-18Si-5Al, and Nb-24Ti-18Si-5Al-5Cr, and ZF4, ZF5 and ZF6 would also suggest that Cr had a detrimental effect on the mechanical behaviour of the oxide scale and its porosity. 

The k_p_ values of the alloys ZF4, ZF6, and ZF9 were at least one order of magnitude lower than those of the alloys Nb-24Ti-18Si-5Al-5Cr-2Mo (2.5 × 10^−11^ mg^2^·cm^−4^·s^−1^), Nb-24Ti-18Si-5Al-5Cr-5Hf-2Mo (4.7 × 10^−11^ mg^2^·cm^−4^·s^−1^), and Nb-24Ti-18Si-5Al-5Cr-5Hf-5Sn-2Mo (1 × 10^−11^ mg^2^·cm^−4^·s^−1^) [48]. The alloy ZF9 had the lowest k_p_ at 800 °C among all the ZF series alloys. Indeed, the oxidation rate constant of ZF9 was one order of magnitude lower than that of the alloys ZF4 and ZF6, and two orders of magnitude lower than that of the alloy ZF5 and the alloy 2 in [32] (5 × 10^−11^ g^2^·cm^−4^·s^−1^) (see above). According to Geng et al. [25,26], the Hf addition in the alloy Nb-24Ti-18Si-5Al-5Cr-5Hf-2Mo had little effect on its oxidation resistance. Even though the volume fraction of Nb_ss_ in the alloy ZF9 could not be measured [31], it was significantly lower compared with the alloys Nb-24Ti-18Si-5Al-5Cr-5Hf-2Mo (about 39.1% [48]) and Nb-24Ti-18Si-5Al-5Cr (about 51% [47]) and lower than that in the alloy ZF6. Thus, the effect of the synergy of Hf and Ge on reducing further the vol % of the Nb_ss_ (see below) must have contributed to the better oxidation behaviour of the alloy ZF9 at 800 °C.

#### 4.1.3. Role of Microstructure

The oxidation resistance of Nb-silicide based alloys is sensitive to the vol % of Nb_ss_ [47,48,49]. The Ge addition in the ZF series alloys resulted to lower vol% of the Nb_ss_ in ZF4 (15.4 ± 3.1 %) [29], ZF5 (16.4 ± 3.8%) [30] and ZF6 (17.3 ± 3.5%) [31] compared with the KZ series alloys Nb-24Ti-18Si-5Cr (48–55% [47]), Nb-24Ti-18Si-5Al-5Cr (48–55% [47]), Nb-24Ti-18Si-6Ta-5Al-5Cr (50–59% [47]), and the JG series alloys Nb-24Ti-18Si-5Al-5Cr-5Mo (34.2% [50]), Nb-24Ti-18Si-5Al-5Cr-2Mo (35.9% [50]), and Nb-24Ti-18Si-5Al-5Cr-5Hf-2Mo (39.1% [24]). The better oxidation behaviour of the alloys ZF4, ZF5, ZF6, and ZF9 at 800 °C compared with the aforementioned KZ and JG series of alloys must be linked with the effect of Ge on the vol % of Nb_ss_. 

There was lower oxygen concentration in the Nb_ss_ in the diffusion zone in ZF4 compared with ZF5, and the diffusion zone in the former was less than that in the latter, in agreement with the data for the alloys Nb-24Ti-18Si-5Cr and Nb-24Ti-8Si-5Al [47]. The oxidation of the alloy ZF4 was better than that of ZF5 but the opposite was the case for the alloys Nb-24Ti-18Si-5Cr and Nb-24Ti-8Si-5Al [47]. Thus, in the presence of Ti and Ge, the Cr addition had a stronger effect in reducing the oxidation rate and the contamination of the microstructure than the Al addition. According to Zelenitsas and Tsakiropoulos [47], the C14-Cr_2_Nb Laves phase is not beneficial for oxidation at 800 °C. In the alloy ZF4, only a very small vol % of this Laves phase was formed [29]. The vol % of Nb_ss_ in ZF4 was slightly lower than that in the alloy ZF5 (see above). The concentrations of Si and Ge in the Nb_ss_ (64.5Nb-24.8Ti-1.9Si-2.0Ge-6.8Cr) in ZF4 prior to oxidation were close to those in the Nb_ss_ (61.7Nb-28Ti-2.2Si-1.9Ge-6.2Al) in ZF5 and the latter was richer in Ti. The diffusivity of oxygen in Nb_ss_ is reduced in the presence of Ti and Cr [45,47]. Thus, the better oxidation behaviour of the alloy ZF4 compared with ZF5 was attributed to the synergy of Cr and Ge in the Nb_ss_ and the vol% of the latter. 

The concentrations of the alloying elements in the Nb_ss_ in the diffusion zone were reduced compared with the composition of the Nb_ss_ in the bulk of the alloys. The Nb_2_O_5_ and TiNb_2_O_7_ were dominant and there was a higher reduction in the concentrations of Nb and Ti in the diffusion zone compared with Al, Cr, Si, and Ge. In the alloys ZF4 and ZF5, the oxygen concentrations in the Nb_5_Si_3_ (≤2.6 at.%) in their diffusion zones were significantly lower compared with those in the Nb_ss_ (about 21.5 at.% and 32 at.%). The oxygen solubility in the Nb_5_Si_3_ alloyed with Al and/or Cr was not affected by Ge.

As was the case for the KZ [47] and JG [25,26,48] series alloys oxidised at 800 °C, micro-cracks parallel to the surface of the scales were observed in the Nb_5_Si_3_ in the diffusion zones of the alloys ZF4 and ZF5. Cracks were also extended into the Nb_ss_ but at a significantly smaller fraction, which would suggest that the oxidised Nb_ss_ was more resistant to cracking than Nb_5_Si_3_. An alternative explanation could be that the higher volume expansion of the oxidised Nb_ss_ imposed tensile stresses on Nb_5_Si_3_ and caused its cracking [25]. 

#### 4.1.4. Scales

The oxides formed on the alloy ZF4 were a mixture of Nb_2_O_5_ and TiNb_2_O_7_, with (probably small amounts of) CrNbO_4_, SiO_2_, and GeO_2_. According to Prokoshikin and Vasil, eva [8], the structure of TiNb_2_O_7_ (Nb_2_O_5_·TiO_2_) is less favourable for the diffusion of oxygen ions than that of Nb_2_O_5_. The CrNbO_4_ (Nb_2_O_5_·Cr_2_O_3_) has the rutile structure [4] with a considerable homogeneity range extending from the stoichiometric composition to a higher Nb_2_O_5_ content in the Nb_2_O_5_-Cr_2_O_3_ system [51]. This oxide was more effective than TiNb_2_O_7_ in reducing the oxidation rate of the alloy Nb-22.5Ti-4.0Hf-15.6Cr-17.3Si-4.8Ge after cyclic oxidation between ambient temperature and 900 °C [52]. The Nb_2_O_5,_ TiNb_2_O_7_ and CrNbO_4_ oxides were also formed in the scale of the alloy Nb-24Ti-18Si-5Cr in which peaks of SiO_2_ were not observed [47].

In the alloy ZF4, the diffraction peaks of the oxides Nb_2_O_5_ and TiNb_2_O_7_ were observed for γ values of 1° to 14° and most of the peaks of SiO_2_ and GeO_2_ were not observed for the γ values of 1° and 2°. Since the depth of X-ray penetration increases with increasing γ, it is suggested that the former two oxides were formed throughout the scale, and the latter two oxides were formed at a later stage. Thus, Nb and Ti had reacted stronger with oxygen than Si and Ge, which is in agreement with [47,48,49,50,51,52]. This was supported by the difference in the compositions of the Nb_ss_ between the diffusion zone and the bulk. The Nb and Ti diffused from the bulk towards the alloy/scale interface where they were consumed to produce the oxides Nb_2_O_5_ and TiNb_2_O_7_. Most diffraction peaks of the CrNbO_4_ were observed at the high glancing angles, which indicated that this oxide was formed in the interior rather than the surface of the scale on ZF4. Thus, in the oxidation of the alloy ZF4 (i) the Nb and Ti reacted strongly with oxygen to form the oxides Nb_2_O_5_ and TiNb_2_O_4_ that continued to grow throughout the oxide scale and (ii) the CrNbO_4_ formed at a later stage of the oxidation process. During oxidation the outward diffusion of the Cr cation was slower than the inward diffusion of oxygen anion through the mixtures of the Nb_2_O_5_ and TiNb_2_O_7_ oxides.

The oxides formed on the alloy ZF5 were a mixture of Nb_2_O_5_, TiNb_2_O_7,_ and AlNbO_4_ with possibly small amounts of SiO_2_ and GeO_2_. The Al addition promoted the formation of AlNbO_4_, which is in agreement with the data for Nb-24Ti-18Si-5Al in which the oxides Nb_2_O_5_, 5Nb_2_O_5_·TiO_2_, 3Nb_2_O_5_·TiO_2_, Nb_2_O_5_·TiO_2_ (TiNb_2_O_7_), and AlNbO_4_ were formed [47]. According to the Nb_2_O_5_-Al_2_O_3_ phase diagram in [51], Al_2_O_3_ is soluble in Nb_2_O_5_, and the AlNbO_4_ (Nb_2_O_5_·Al_2_O_3_) extends from the stoichiometric composition up to 70 mol % Al_2_O_3_. The AlNbO_4_ is beneficial in reducing the oxidation rate as the (Nb,Al)_2_O_5_ impedes the diffusion of oxygen [8]. The GXRD results of the alloy ZF5 suggested that the AlNbO_4_ was formed throughout the oxide scale as most of the diffraction peaks of this phase were observed at all glancing angles. 

The oxides formed on the alloys ZF4 and ZF5 were also observed on the alloys ZF6 and ZF9 with HfO_2_ forming in the latter. The evidence for SiO_2_ and GeO_2_ in the scales of the latter two alloys was not strong, in agreement with [32,47,52]. 

### 4.2. Oxidation at 1200 °C

A change from parabolic to linear oxidation kinetics happens when the scale gets partially cracked or micro-cracks appear at the scale/substrate interface, which leads to direct access of the oxidising atmosphere to the substrate resulting in fast oxidation rates [53]. The oxidation of the alloys ZF4, ZF5, ZF6, and ZF9 at 1200 °C followed parabolic kinetics at an initial stage and changed to linear oxidation. This behaviour has been reported before for Nb-silicide based alloys, for example for the alloy Nb-24Ti-18Si-5Cr-5Al-5Hf-5Sn-2Mo [25]. Thus, it is suggested that before 35 h for the alloys ZF4 and ZF5 and 20 h for the alloys ZF6 and ZF9 the oxide growth process was governed by the diffusion of ions or electrons through the initially formed scale, and then these alloys suffered from cracking of their scales that resulted to a change in oxidation kinetics. 

In the first 20 h, the alloy ZF4 followed parabolic kinetics with mass change lower than the alloy ZF5. This supports the argument for the synergy of Cr and Ge having a stronger effect on oxidation than that of Al and Ge. After 35 h, the alloy ZF4 followed linear kinetics with higher oxidation rate compared with the alloy ZF5. Assuming that the change of the oxidation kinetics from parabolic to linear was caused by the generation of cracks in the scale, the higher oxidation rate of the alloy ZF4 after 35 h was attributed to Cr having a stronger effect than Al on causing deterioration of the mechanical behaviour of the scale. 

The better oxidation resistance of the alloy ZF6 compared with the alloys ZF4 and ZF5 would suggest that in the presence of Ti and Ge the synergy of Cr and Al was more beneficial to oxidation resistance than the individual effect of Cr or Al. In contrast to the oxidation at 800 °C, the alloys ZF6 and ZF9 exhibited the same oxidation behaviour at 1200 °C. Thus, in the presence of Cr and Al, the Hf addition had little effect on the oxidation at 1200 °C, which is in agreement with [54]. The parabolic rate constants of the alloys ZF6 and ZF9 were lower than that of Nb-26Ti-12.6Si-6.7Cr-5Ge-1.9Al-1.9Hf-0.5Sn-(B,Ce,Fe) (alloy 2 in [32]) at 1200 °C (about 3.5 × 10^−9^ g^2^·cm^−4^·s^−1^), which would suggest that the synergy of Fe, Sn, and B with Ti, Al, Cr, Hf, and Ge in this alloy was detrimental to its oxidation behaviour at 1200 °C (the same was the case at 800 °C, see above). The parabolic rate constants of the alloys ZF6 and ZF9 were also one order of magnitude lower than that of the alloy Nb-24Ti-16Si-6Cr-6Al-2Hf (about 7 × 10^−9^ g^2^·cm^−4^·s^−1^) [55], which supports the case that the synergy of Ge with Ti, Al, and Cr is beneficial for oxidation resistance. 

Comparison of the total mass changes after exposure for 100 h at 1200 °C of the alloys ZF4 (59 mg·cm^−2^) with Nb-24Ti-18Si-5Cr (140 mg·cm^−2^ after 65 h [47]), ZF5 (55 mg·cm^−2^) with Nb-24Ti-18Si-5Al (100 mg·cm^−2^ after 65 h [47]), ZF6 (40 mg·cm^−2^) with Nb-24Ti-18Si-5Al-5Cr (95 mg·cm^−2^ [47]), and ZF9 (41 mg·cm^−2^) with Nb-24Ti-18Si-5Al-5Cr-5Hf-2Mo (119 mg·cm^−2^ [25,48]) and Nb-24Ti-18Si-5Al-5Cr-5Hf-5Sn-2Mo (89 mg·cm^−2^ [26,48]) confirms the beneficial effect of Ge on the oxidation resistance of the ZF series Nb-silicide based alloys, as was the case for the oxidation at 800 °C. 

The oxides present in the scales at 1200 °C were the same as those that formed at 800 °C and in agreement with [25,32,47]. Spallation of the scales occurred in all the ZF series alloys at 1200 °C, as was the case for the alloys Nb-24Ti-18Si-5Al-5Cr-2Mo and Nb-24Ti-18Si-5Al-5Cr-5Hf-2Mo [25]. The dominant oxides at 1200 °C were Nb_2_O_5_ and TiNb_2_O_5_. The scale spallation was attributed to stress generation in the scale during oxide growth [53]. The stresses started accumulating with the oxide growth process(es) and were released when the scale thickness was unable to bear the increased stress. The release of stress could either be attributed to cracking in the scale or creep of the substrate. Both of these factors will lead to scale spallation. The ratio of the molar volume of metal to the molar volume of the oxide formed on it (Pilling Bedworth Ratio, PBR) is an important factor responsible for the accumulation of stress. If there is a large difference in the two, then the oxide has a tendency to spall. For good adhesion of the scale, the PBR should be equal to or close to 1. The Nb_2_O_5_ has PBR = 2.68 [53], which explains the spallation of the scales when a critical volume of the oxide was formed.

The metal recession rate (the ratio of scale thickness to oxidation time) of the alloy ZF6 was about 3.95 µm h^−1^ at 1200 °C and was the smallest among all ZF series alloys. Compared with the metal recession rates of the alloys Nb-25Ti-8Hf-2Cr-2Al-16Si (14 µm h^−1^), Nb-26Ti-4Hf-2Cr-2Al-16Si (6–14 µm h^−1^), and Nb-28Ti-2Cr-2Al-16Si (5–8 µm h^−1^) at 1200 °C [55], the oxidation resistance of the alloy ZF6 was better. 

### 4.3. Comparison with Refractory Metal HEAs

The alloying behaviour of Nb-silicide based alloys and of the most important phases in their microstructures, and the properties of these alloys and their phases can be described by the parameters δ (related to atomic size), Δχ (related to electronegativity), and number of valence electrons per atom filled into the valence band (VEC) [4,5,6,7,14,56]. According to the alloy design methodology NICE [4] the trends of VEC and δ for oxidation resistance are opposite and alloy design/selection should decrease the former and increase the latter. NICE was recently used to design non-pesting and alumina scale forming Nb-Ti-Si-Al-Hf HEA alloys [57,58]. 

**Figure 14:**
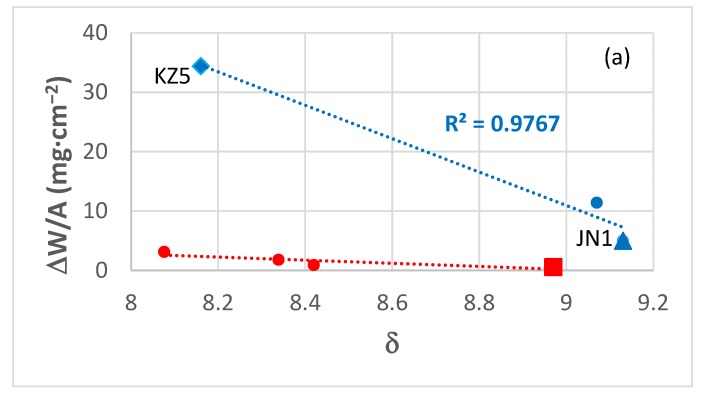

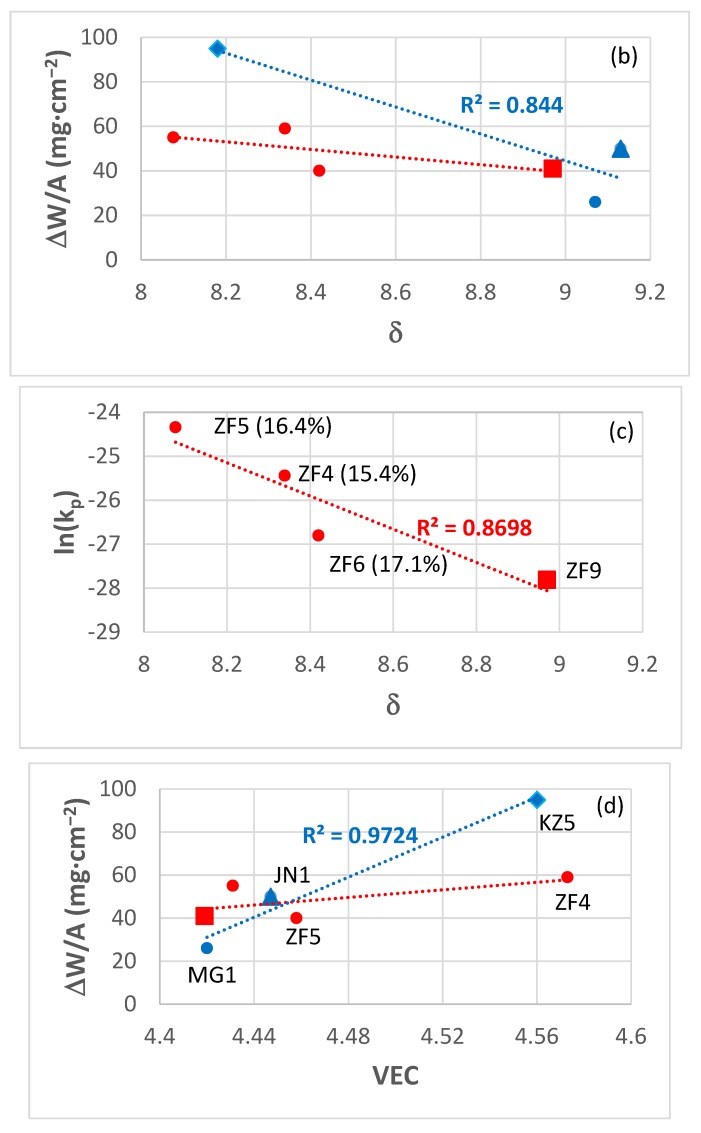
Plots of ΔW/A versus the parameters δ and VEC for alloys with/out Ge addition. Data in (**a**) and (**c**) for 800 °C and in (**b**) and (**d**) for 1200 °C. In (**a**), (**b**), and (**d**) data for the alloys KZ5 (Nb-24Ti-18Si-5Al-5Cr [59]), MG1 (Nb-24Ti-18Si-5Al-5Hf [60]) and JN1 (Nb-24Ti-5Al-5Cr-5Hf [60]) is shown in blue. In all figures, the HEA (ZF9) is shown by the red square. The vol % Nb_ss_ in the alloys ZF4, ZF5, and ZF6 in the bulk of their buttons, from which the oxidation specimens were selected, is shown in (**c**).

The alloy ZF9 could be considered as a Refractory Metal (RM) bcc solid solution + intermetallic High Entropy Alloy (HEA) or Complex Concentrated Alloy (CCA) [31] because it has “principal elements with the concentration of each element being between 35 and 5 at.%”. Figure 14 shows plots of mass change (ΔW/A) versus the parameter δ for 800 °C (Figure 14a) and 1200 °C (Figure 14b) and the parameter VEC for 1200 °C (Figure 14d) and of ln(k_p_) versus the parameter δ for 800 °C (Figure 14c) for alloys without Ge addition (blue data, alloys KZ5=Nb-24Ti-18Si-5Al-5Cr [59], MG1=Nb-24Ti-18Si-5Al-5Hf, and JN1=Nb-24Ti-18Si-5Al-5Cr-5Hf [60]) and for the alloys ZF4, ZF5, ZF6, and ZF9 (red data). Figure 14 shows improved oxidation behaviour at both temperatures of the alloys with/out Ge as the parameters δ and VEC respectively increase and decrease. Note (a) that data about the vol % Nb_ss_ in alloys with Ge is included in Figure 14, (b) that there is a better correlation between improved oxidation (lower KP) and the parameter δ (see below) than vol % Nb_ss_, and (c) that with the addition of Ge to ZF6 or ZF9 the ΔW/A and VEC decreased at 1200 °C (compare KZ5 and ZF6, and JN1 and ZF9 in Figure 14d) and 800 °C (figure not shown).

Direct comparison of the alloy ZF9 with the scarce data about the oxidation of RM HEAs is not possible because (i) oxidation has been evaluated at different temperature(s), (ii) isothermal oxidation was evaluated in some studies and cycling oxidation in others, (iii) different atmosphere and duration were used, (iv) different alloying elements were present in the constitution of the HEAs, and (v) the HEAs had different microstructures.

The isothermal oxidation of the RM HEA NbCrMo_0.5_Ta_0.5_TiZr (two bcc solutions + Laves phase) at 1000 °C for 100 h in dry air was studied by Senkov et al [61]. The mass change was 120 mg·cm^−2^ (compared with 0.55 and 41 mg·cm^−2^ of ZF9 respectively at 800 and 1200 °C), a continuous scale formed with a rapid increase in weight during the first 10 h of holding at 1000 °C and then the rate of weight increase continuously decreased with an increase in the oxidation time (similar behaviour was exhibited by ZF9), six plate-like solid pieces of the scale, corresponding to the six rectangular surfaces of the oxidation sample separated from the remaining sample core (similar to the alloys ZF4, ZF5, and ZF6 but not ZF9 where separation of the scale occurred only from three surfaces), the thickness of the scale was 1750 μm (about four times that of ZF9) and numerous oxides formed in the scale of which only CrNbO_4_ and Nb_2_O_5_ were common with ZF9. 

Considering the values of the parameters δ and VEC of NbCrMo_0.5_Ta_0.5_TiZr, 6.79 and 4.906 respectively, and the data in Figure 14, it is not surprising that the oxidation of this RM HEA at 1000 °C was inferior to that of ZF9 at 800 and 1200 °C. Actually, if the data for the non-Ge containing alloys in Figure 14a and b are considered (blue data), the arithmetic average of the mass changes calculated for δ = 6.79 at 800 and 1200 °C is 125.6 mg·cm^−2^. In other words, the composition of the HEA NbCrMo_0.5_Ta_0.5_TiZr “assured” poor oxidation because its parameters δ and VEC are on the wrong side of the required ranges. 

Liu et al studied the isothermal oxidation in air at 1300 °C for 20 h of the RM HEAs NbCrMoTiAl_0.5_ (RM_ss_ + βTi_ss_, ΔW/A = 150 mg·cm^−2^, k_l_ = 2.09 × 10^−6^ mg·cm^−2^·s^−1^, δ = 5.14, VEC = 5.0122), NbCrMoVAl_0.5_ (RM_ss_, ΔW/A = 350 mg·cm^−2^, k_l_ = 4.79 × 10^−6^ mg·cm^−2^·s^−1^, δ = 4.75, VEC = 5.249), NbCrMoTiVAl_0.5_ (RM_ss_ + βTi_ss_, ΔW/A = 275 mg·cm^−2^, k_l_ = 3.67 × 10^−6^ mg·cm^−2^·s^−1^, δ = 5, VEC = 5.0258) and NbCrMoTiVAl_0.5_Si_0.3_ (RM_ss_ + (Nb,Ti)_5_Si_3_, ΔW/A = 200 mg·cm^−2^, k_l_ = 2.73 × 10^−6^ g·cm^−2^·s^−1^, δ = 5.97, VEC = 4.9622) [62]. The microstructures of these RM HEAs were not the same, one was a bcc solid solution, another was a bcc solid solution + Nb_5_Si_3_ silicide and another two bcc solid solutions and all were different compared with ZF9. The poor oxidation of these alloys was to be expected considering that their VEC and δ values are in the wrong side of the required ranges. Actually, if their δ values are plotted versus ΔW/A or ln(k_l_) and their VEC values are plotted versus ΔW/A the data shows improved oxidation, meaning lower ΔW/A and lower k_l_ as δ increases and VEC decreases.

No data for the oxidation of the RM HEAs in the pest regime was given in [61,62]. Most likely, these RM HEAs would pest, unlike ZF9 which did not suffer from catastrophic pest oxidation.

The cyclic oxidation in air (?) at 1100 °C for a total time of about 500 h of the alloy 35.8Nb-22.5Ti-4.0Hf-15.6Cr-17.3Si-4.8Ge was studied by Chan [52]. Its microstructure contained 73% Nb_5_Si_3_ and 27% NbCr_2_ Laves phase (i.e., no Nb_ss_ was present) and the oxides CrNbO_4_, 3Nb_2_O_5_·TiO_2_, Ti_2_Nb_10_O_29,_ and GeO_2_ (minor component) were detected in the scale by X-ray diffraction. Both the mass change of the test specimens and the mass of spalled scale were very small after the cyclic oxidation. This alloy also satisfies the definition of HEAs and has δ = 9.31 and VEC = 4.67, respectively larger and smaller than the values of the same parameters of the alloys studied by Senkov et al [61] and Liu et al [62] (see above). Note that the oxidation-resistant alloy 34.9Nb-24.7Ti-18.5Si-4.9Al-4.9Cr-5.3Hf-5Sn-1.8Mo [48] has δ = 9.57 and VEC = 4.434. The available oxidation data for RM HEAs and Nb-silicide based alloys confirms the improvement of oxidation resistance with increasing δ and decreasing VEC, in agreement with NICE [4].

## 5. Conclusions

A preliminary study of the isothermal oxidation in air at 800 and 1200 °C for 100 h of the as-cast alloys Nb-18Si-5Ge (ZF1), Nb-18Si-10Ge (ZF2), Nb-24Ti-18Si-5Ge (ZF3), Nb-24Ti-18Si-5Cr (ZF4), Nb-24Ti-18Si-5Al (ZF5), Nb-24Ti-18Si-5Al-5Cr-5Ge (ZF6) and Nb-24Ti-18Si-5Al-5Cr-5Ge-5Hf (ZF9) was reported. Only the alloys ZF1, ZF2, and ZF3 suffered from catastrophic pest oxidation at 800 °C. The Ge addition substantially improved the oxidation resistance of the other alloys both at 800 °C and 1200 °C. The alloys ZF9 and ZF6 exhibited the best oxidation behaviour at 800 °C and 1200 °C, respectively. 

The alloys were compared with Nb-silicide based alloys of similar compositions without Ge and Hf additions and the alloy ZF9 with RM HEAs. Compared with the former alloys, the addition of Ge reduced the vol % of the Nb_ss_. Compared with the latter alloys, the superior oxidation behaviour of the alloy ZF9 was attributed to its higher and lower values respectively of the parameters δ and VEC. 

The oxidation of the alloys ZF4, ZF5, ZF6, and ZF9 followed parabolic kinetics at 800 °C. At 1200 °C there was spallation of their scales. Their oxidation had followed parabolic kinetics in the early stages and linear kinetics at longer times. 

Diffusion zones were formed below the scale/substrate interface only in the alloys ZF4 and ZF5. In these alloys cracks parallel to the scale/substrate interface were observed in the Nb_5_Si_3_. 

The Nb_ss_ and Nb_5_Si_3_ in the diffusion zones were contaminated by oxygen, the former more severely than the latter. The Nb_ss_ and Nb_5_Si_3_ were not contaminated by oxygen in the bulk of the alloys ZF4, ZF5, ZF6, and ZF9. The alloying with Cr was more effective in reducing the oxygen diffusivity compared with Al, but the Cr addition had adverse effect on the mechanical behaviour of the scales.

In the scales that formed on the alloys ZF4, ZF5, ZF6, and ZF9 at 800 °C and 1200 °C the oxides Nb_2_O_5_ and TiNb_2_O_7_ were present, GeO_2_ was observed in all scales and SiO_2_ in the scales of the alloys ZF4, and ZF5, CrNbO_4_ in the scales of the alloys ZF4, ZF6, and ZF9 and AlNbO_4_ in the scales of the alloys ZF5, ZF6, and ZF9. 

## Figures and Tables

**Table 1:** Phases in the microstructures of the as-cast alloys of this study.

Alloy	Phases	Ref.
ZF1	Nb_ss_, βNb_5_(Si,Ge)_3_, (Nb_ss_ + Nb_5_(Si,Ge)_3_)_eutectic_	[27]
ZF2	Nb_ss_, βNb_5_(Si,Ge)_3_, (Nb_ss_ + Nb_5_(Si,Ge)_3_)_eutectic_	[27]
ZF3	Nb_ss_, β(Nb,Ti)_5_(Si,Ge)_3_, (Ti,Nb)_5_(Si,Ge)_3_, (Nb,Ti)_3_(Si,Ge)	[28]
ZF4	Nb_ss_, βNb_5_(Si,Ge)_3_, (Nb_ss_ + Nb_5_(Si,Ge)_3_)_eutectic_, C14-NbCr_2_	[29]
ZF5	Nb_ss_, βNb_5_(Si,Ge,Al)_3_, (Nb_ss_ + Nb_5_(Si,Ge,Al)_3_)_eutectic_	[30]
ZF6	Nb_ss_, βNb_5_(Si,Ge,Al)_3_, (Nb_ss_ + Nb_5_(Si,Ge,Al)_3_)_eutectic_, C14-NbCr_2_	[31]
ZF9	Nb_ss_, βNb_5_(Si,Ge,Al)_3_, C14-NbCr_2_	[31]

**Table 2:** Total mass changes and oxidation rate constants of the alloys at 800 °C.

Alloy Code	Mass Change (mg·cm^−2^)	*k_l_* (g·cm^−2^·s^−1^)	*k_p_* (g^2^·cm^−4^·s^−1^)	Scale Thickness (μm)
ZF1	47.0 (0.67 h)	1.9 × 10^−5^ (0.67 h)	—	
ZF2	33.2 (0.67 h)	1.4 × 10^−5^ (0.67 h)	—	
ZF3	9.6 (10 h)	2.7 × 10^−7^ (10 h)	—	
ZF4	1.79 (100 h)	—	8.9 × 10^−12^ (100 h)	50
ZF5	3.11 (100 h)	—	2.7 × 10^−11^ (100 h)	13
ZF6	0.91 (100 h)	—	2.3 × 10^−12^ (100 h)	8
ZF9	0.55 (100 h)	—	8.4 × 10^−13^ (100 h)	1

**Table 3:** The Electron Microprobe Analysis (EPMA) data of the constituent phases in the diffusion zone and bulk of the oxidised alloy ZF4.

Area	Phase	Composition (at.%)					
		Nb	Ti	Si	Ge	Cr	O
**Diffusion Zone**	Nb_ss_	51.5	18.2	1.6	1.4	5.7	21.6
	Nb_5_Si_3_	44.4	18.4	26.8	6.9	1.1	2.4
**Bulk**	Nb_ss_	69.2	21.6	1.6	1.8	5.8	0.0
	Nb_5_Si_3_	45.7	18.6	27.1	7.5	1.1	0.0

**Table 4:** The composition of constituent phases in the diffusion zone and bulk in the oxidised alloy ZF5.

Area	Phase	Composition (at.%)					
		Nb	Ti	Si	Ge	Al	O
**Diffusion Zone**	Nb_ss_	43.1	17.9	1.3	1.3	4.4	32.0
	Nb_5_Si_3_	44.1	17.7	25.6	6.9	3.1	2.6
**Bulk**	Nb_ss_	60.7	29.5	1.7	2.0	6.1	0.0
	Nb_5_Si_3_	42.6	22.3	23.9	7.2	4.0	0.0

**Table 5:** The composition (at.%) of the constituent phases in the bulk of the oxidised alloy ZF6.

Area	Phase	Nb	Ti	Si	Ge	Cr	Al	O
**Bulk**	Nb_ss_	53.4	27.3	1.5	1.4	8.7	7.7	0.0
	Nb_5_Si_3_	44.3	20.5	23.7	7.8	1.2	2.5	0.0

**Table 6:** The composition (at.%) of the constituent phases in the bulk in the oxidised alloy ZF9.

Area	Phase	Nb	Ti	Si	Ge	Cr	Al	Hf	O
**Bulk**	Nb_ss_	44.4	29.1	1.5	1.1	13.4	7.5	3.0	0
	Nb_5_Si_3_	38.4	19.6	25.2	7.1	1.2	3.6	4.9	0
	Hf-rich Nb_5_Si_3_	24.7	26.7	23.7	7.4	3.1	4.1	10.3	0

**Table 7:** Oxidation rate constants of the alloys at 1200 °C.

Alloy Code	*k_p_* (g^2^·cm^−4^·s^−1^)	*k_l_* (g·cm^−2^·s^−1^)
ZF4	6.7 × 10^−9^ (≤35 h)	1.3 × 10^−7^ (>35 h)
ZF5	6.4 × 10^−9^ (≤35 h)	1.1 × 10^−7^ (>35 h)
ZF6	2.0 × 10^−9^ (≤20 h)	9.7 × 10^−8^ (>20 h)
ZF9	1.8 × 10^−9^ (≤20 h)	1.0 × 10^−7^ (>20 h)

**Table 8:** The thickness of the spalled oxide scales formed on the oxidised alloys after exposure for 100 h at 1200 °C.

Alloy Code	Thickness of Oxide Scale (µm)
ZF4	682 ± 20
ZF5	686 ± 32
ZF6	395 ± 12
ZF9	465 ± 27
